# Elucidating Chiral Resolution of Aromatic Amino Acids Using Glycopeptide Selectors: A Combined Molecular Docking and Chromatographic Study

**DOI:** 10.3390/ijms25169120

**Published:** 2024-08-22

**Authors:** Dehbiya Gherdaoui, Madiha Melha Yahoum, Selma Toumi, Sabrina Lekmine, Sonia Lefnaoui, Ouided Benslama, Rachida Bouallouche, Hichem Tahraoui, Mohammad Shamsul Ola, Ahmad Ali, Jie Zhang, Abdeltif Amrane

**Affiliations:** 1 Laboratory of Biomaterials and Transport Phenomena (LBMPT), New Urban Pole, Medea University, Medea 26000, Algeria; 2 Higher Normal School, Laboratory of Research on Bio-Active Products and Valorization of Biomasse, Old-Kouba, Algiers 16050, Algeria; 3 Materials and Environmental Laboratory (LME), University of Medea, New Urban Pole, Medea 26000, Algeria; 4 Biotechnology, Water, Environment and Health Laboratory, Abbes Laghrour University, Khenchela 40000, Algeria; 5 Laboratory of Natural Substances, Biomolecules and Biotechnological Applications, Department of Natural and Life Sciences, Larbi Ben M’Hidi University, Oum El Bouaghi 04000, Algeria; 6 Reaction Engineering Laboratory, Faculty of Mechanical and Process Engineering, University of Science and Technology Houari Boumediene, BP32 El Alia, Bab Ezzouar, Algiers 16111, Algeria; 7 Laboratory of Chemical Process Engineering, Department of Process Engineering, Faculty of Technology, Ferhat Abbas University, Setif-1, Setif 19000, Algeria; 8 National Higher School of Chemistry of Rennes, Scientific Research National Center (CNRS), Rennes Institute of Chemical Sciences—Mixed Research Unit (ISCR—UMR6226), Rennes University, 35000 Rennes, France; 9 Department of Biochemistry, College of Science, King Saud University, Riyadh 11451, Saudi Arabia; 10 Department of Life Sciences, University of Mumbai, Vidyanagari, Mumbai 400098, India; 11 School of Engineering, Merz Court, Newcastle University, Newcastle upon Tyne NE1 7RU, UK

**Keywords:** chiral resolution, molecular docking, aromatic amino acids, elution order, glycopeptide selector

## Abstract

An asymmetric synthesis is a favorable approach for obtaining enantiomerically pure substances, but racemic resolution remains an efficient strategy. This study aims to elucidate the chiral resolution of aromatic amino acids and their elution order using glycopeptides as chiral selectors through molecular docking analysis. Chiral separation experiments were conducted using Vancomycin as a chiral additive in the mobile phase (CMPA) at various concentrations, coupled with an achiral amino column as the stationary phase. The Autodock Vina 1.1.2 software was employed to perform molecular docking simulations between each enantiomer (ligand) and Vancomycin (receptor) to evaluate binding affinities, demonstrate enantiomeric resolution feasibility, and elucidate chiral recognition mechanisms. Utilizing Vancomycin as CMPA at a concentration of 1.5 mM enabled the separation of tryptophan enantiomers with a resolution of 3.98 and tyrosine enantiomers with a resolution of 2.97. However, a poor chiral resolution was observed for phenylalanine and phenylglycine. Molecular docking analysis was employed to elucidate the lack of separation and elution order for tryptophan and tyrosine enantiomers. By calculating the binding energy, docking results were found to be in good agreement with experimental findings, providing insights into the underlying mechanisms governing chiral recognition in this system and the interaction sites. This comprehensive approach clarifies the complex relationship between chiral discrimination and molecular architecture, offering valuable information for creating and improving chiral separation protocols.

## 1. Introduction

Over the past 30 years, the development of efficient and reliable chiral separation methods has become essential. Chirality is considered an essential issue in different areas of the modern world, especially in the field of drug research and development. Regulatory agencies continue to emphasize the importance of enantiomeric purity and its critical impact on the safety and effectiveness of drug substances [1]. For a pair of enantiomers, the pharmacological properties are far from being similar and can be completely different when they interact with the human body, which is itself an environment made up of a series of chiral structures like amino acids [1].

Amino acids, as well as proteins and other derivatives that they form, are involved in all biological processes of living organisms, plants, and animals. Enantiopure amino acids serve as essential building blocks across a diverse range of industries, including pharmaceuticals, agrochemicals, and the production of food and fragrances. While L-amino acids are the fundamental components of proteins, their D-enantiomers have garnered significant interest due to their diverse applications in antibiotics, chemotherapeutics, immunosuppressants, deodorants, fluorescent DNA markers, sweeteners, pesticides, and many other fields [2,3]. The importance of D-amino acids is underscored by their inclusion in the structure of approximately 5% of the top 200 brand-name drugs based on retail sales in 2018 [4,5]. It is noteworthy that all naturally occurring amino acids predominantly exist in the L-isomer (levorotatory) form, which contrasts with the prevalent presence of D-isomers (dextrorotatory) in natural sugars (carbohydrates) [6]. This distinction between the isomers of amino acids and sugars highlights the unique stereochemical preferences observed in nature and emphasizes the importance of understanding and utilizing both enantiomers in various applications.

**Figure 1:**
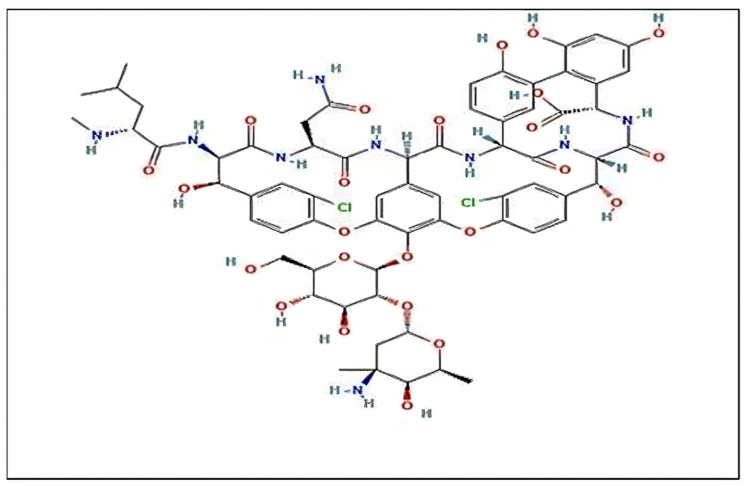
Vancomycin structure.

Dalglish outlines a crucial criterion for successful enantiomeric separation, emphasizing the necessity for structural complementarity between the enantiomers and the chiral selector, with a minimum requirement of three interaction sites [7,8]. Among the arsenal of chiral selectors, macrocyclic antibiotics stand out as exceptionally effective agents in various chiral separation methodologies. They are renowned for their ability to yield consistent results across a diverse array of applications [9]. The efficacy of these macrocyclic antibiotics, such as Vancomycin, stems from their intricate molecular architecture, which is characterized by a rich diversity of functional groups, cavities, and numerous stereogenic centers, as depicted in Figure 1. This structural complexity provides a fertile ground for a plethora of interaction modalities. These include hydrogen bonding, electrostatic interactions, π–π interactions, inclusion phenomena, steric effects, and dipole stacking [10,11,12]. The combination of these various interaction types allows macrocyclic antibiotics to achieve high selectivity and efficiency in recognizing and separating chiral molecules, making them invaluable tools in the field of chiral separation.

Molecular docking stands as a fundamental technique in the realm of structure-based drug design, playing a pivotal role in simulating molecular interactions and predicting binding modes and affinities between receptors and ligands [13]. Over the past few years, this technology has garnered significant attention and utilization, particularly in the research domain focusing on chirality in drug design [14]. The efficacy of molecular docking lies in its robust ability to model the interactions between enantiomer pairs and the active sites of chiral selectors [15]. This enables the prediction of energy landscapes, as well as the geometric configurations of selector-selected bindings [16]. Such capabilities are immensely valuable for a detailed elucidation of the complex process of chiral recognition [17,18]. By offering insights into both successful and unsuccessful resolution outcomes, molecular docking aids significantly in determining elution orders [19]. This comprehensive understanding is crucial for advancing chiral separation techniques and optimizing the development of enantiomerically pure drugs.

Numerous studies have employed docking simulations to elucidate chiral resolution mechanisms and determine the chiral recognition between a chiral selector as a receptor and chiral samples as ligands. These investigations have consistently demonstrated congruence between the results of docking simulations and experimental outcomes [7,16,17]. The utilization of docking simulations in these studies has provided valuable insights into how chiral selectors interact with various enantiomers, revealing intricate details of binding affinities, interaction energies, and the spatial orientation of the molecules involved. By comparing the computational predictions with empirical data, researchers have been able to validate and refine their theoretical models, ensuring that the docking simulations accurately reflect the real-world behavior of chiral systems. This alignment between simulated and experimental results underscores the reliability and robustness of docking simulations as a powerful tool in the study of chiral resolution mechanisms. Furthermore, the consistent findings across multiple studies highlight the effectiveness of this approach in enhancing our understanding of the molecular interactions that govern chiral recognition. 

The mechanism underlying the separation of enantiomers is ascribed to the formation of transient diastereomeric complexes between the chiral select and chiral selector. The efficacy of enantioseparation is contingent upon the energy difference of formation between these transient diastereomeric complexes; a greater energy disparity signifies superior enantioseparation [18,20]. This energy difference plays a crucial role because it determines how selectively the chiral selector can distinguish between the enantiomers, thereby influencing the overall effectiveness of the separation process. In essence, the larger the energy difference, the more efficiently the chiral selector can separate the enantiomers, resulting in a higher degree of purity for the separated compounds. This fundamental concept is critical for optimizing chiral separation techniques, as it guides researchers in the selection and design of chiral selectors that are capable of maximizing the energy difference and thereby improving the overall separation efficiency.

Moreover, docking simulations offer a detailed understanding of the molecular interactions governing chiral recognition. They unveil the intricate interplay of hydrogen bonding, hydrophobic interactions, and π–π stacking between the chiral selector and enantiomers [21], shedding light on the factors influencing chiral resolution. Additionally, computational studies can predict the elution order of enantiomers and elucidate the structural basis for chiral discrimination [22], thereby guiding the design of optimized chromatographic conditions for enhanced resolution. The integration of docking simulations with experimental data not only validates theoretical models but also enables the refinement of chiral separation strategies, facilitating advancements in enantioselective chromatography.

**Figure 2:**
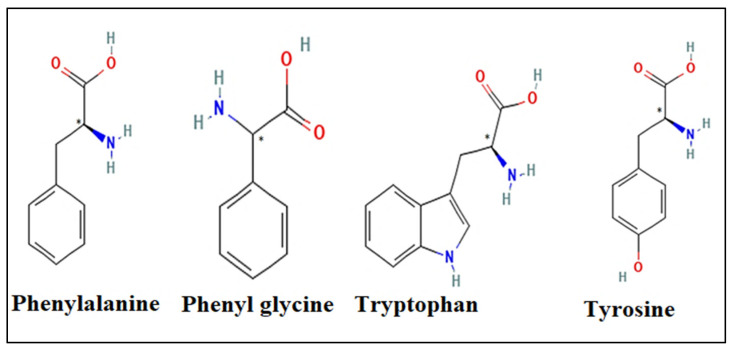
Aromatic amino acids structure.

The primary objective of this study was to elucidate the experimental outcomes concerning the chiral resolution of aromatic amino acids, specifically phenylalanine, phenylglycine, tryptophan, and tyrosine (Figure 2), utilizing glycopeptide as a chiral selector. This investigation employed molecular docking studies conducted through the utilization of AutoDockVina 1.1.2, a molecular modeling software. The aim was to comprehensively analyze all enantiomers against different targets, discerning their binding affinities. Through this analysis, this study sought to unravel the intricate chiral recognition mechanisms at play and demonstrate the feasibility of enantiomeric resolution. By discerning the factors contributing to both successful and unsuccessful resolutions for each sample, this research aimed to provide insights crucial for understanding and optimizing chiral separation methodologies.

## 2. Results and Discussion 

Chromatographic analysis was conducted to assess chiral resolution utilizing Vancomycin in a mobile phase consisting of potassium dihydrogen phosphate (50 mM, pH 6.0) and 2-propanol (50:50, *v*/*v*), operating at a flow rate of 0.8 mL/min. The separation was performed on an NH_2_ column (250 mm × 4.6 mm, 5 µm) coupled with a PDA detector. Table 1 provides a comprehensive overview of all sample results.

From the results presented in Table 1, it is evident that the mixture of phenylalanine enantiomers could not be effectively separated when vancomycin was introduced into the mobile phase composed of potassium dihydrogen phosphate (50 mM, pH 6.0) and 2-propanol (50:50, *v*/*v*), operating at a flow rate of 0.8 mL/min on an NH_2_ column (250 mm × 4.6 mm, 5 µm) coupled with a PDA detector. Although a slight alteration in the retention time of the racemic mixture was observed upon the addition of Vancomycin, no clear trend emerged in the retention times, and resolution was not achieved. This outcome is consistent with findings reported by Dániel et al. [23], who similarly encountered challenges in separating racemic phenylalanine when utilizing Vancomycin as a chiral selector. The poor resolution observed can be attributed to the lack of interaction between the Vancomycin chiral selector and the phenylalanine enantiomers [11,24]. This lack of interaction suggests that the binding affinities and the formation of transient diastereomeric complexes are not adequately strong to achieve effective enantioseparation. Consequently, the chiral selector fails to distinguish between the phenylalanine enantiomers, resulting in a diminished resolution. This issue underscores the importance of selecting a chiral selector with appropriate functional groups and structural characteristics that can form stable and distinct complexes with the target enantiomers.

Similarly, the chromatographic conditions applied proved inadequate for separating the enantiomeric mixture of phenylglycine, as evidenced by the absence of any significant change in the retention time of the racemic mixture upon the addition of Vancomycin to the mobile phase. These results further underscore the absence of interaction between the Vancomycin chiral selector and the enantiomers of the racemic mixture [25]. Notably, while other chiral selectors have demonstrated high selectivity in separating enantiomers, Vancomycin failed to achieve similar results [26,27].

**Figure 3:**
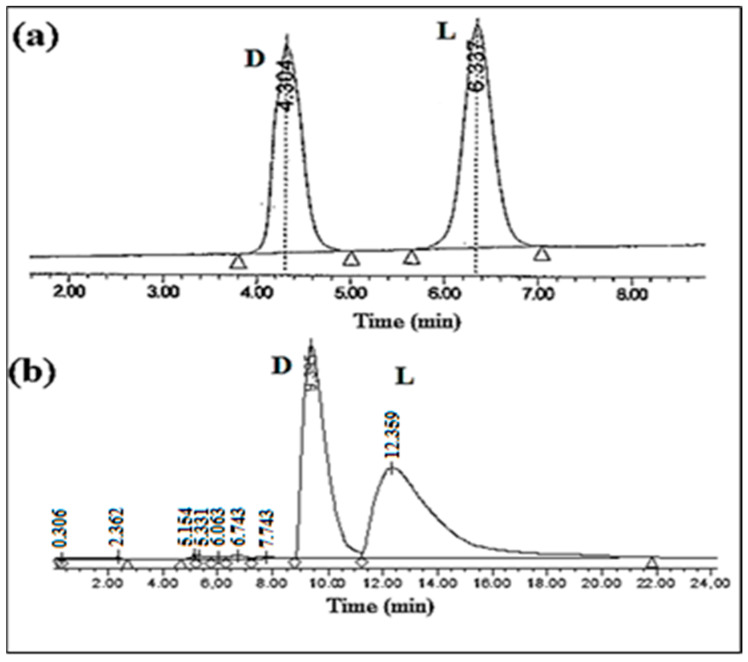
Chromatograms of enantioseparation using vancomycin as a CMPA (1.5 mM), with pH 6.0 phosphate buffer 0.05 M/2-propanol. (**a**) Tryptophan, (**b**) Tyrosine.

In contrast, a breakthrough was achieved in resolving the optical isomers of D,L-tryptophan under the provided chromatographic conditions. Remarkably, this represents the first instance of achieving such a resolution with these specific chromatographic parameters. With an analysis time of less than 15 min, as presented in Figure 3, notable selectivity was observed for tryptophan, particularly when utilizing a Vancomycin concentration of 0.5 mM in the mobile phase, with the D isomer eluting prior to the L form.

Further investigation revealed that the enantioseparation of tryptophan racemic amino acid significantly improved with increasing Vancomycin concentration in the mobile phase. The highest resolution factor (Rs) was achieved at a Vancomycin concentration of 1.5 mM, highlighting the remarkable efficacy of Vancomycin as a chiral selector in facilitating the separation of tryptophan enantiomers. This successful resolution can be attributed to a diverse range of interactions between Vancomycin and tryptophan, encompassing hydrogen bonding, π–π interactions, dipole-dipole interactions, and ionic interactions, which vary depending on the specific experimental conditions applied [9,28]. This comprehensive understanding of the molecular interactions between Vancomycin and tryptophan underscores the versatility and effectiveness of Vancomycin as a chiral selector in enantioseparation processes.

Similarly, it was observed that the chromatographic conditions exerted a significant impact on the enantioseparation of tyrosine (Figure 3). Notably, when employing 1.5 mM of vancomycin in the mobile phase, the enantiomers of tyrosine were effectively separated, achieving a selectivity of 1.50 and a resolution of 2.97. Moreover, the selectivity demonstrated a notable increase with higher concentrations of vancomycin in the mobile phase. The chiral recognition between the vancomycin chiral selector and the tyrosine enantiomers was facilitated by hydrophobic interactions between vancomycin and the tyrosine enantiomers, alongside interactions with the NH_2_ group of the amino column. Furthermore, the presence of the hydroxyl group in the side chain of tyrosine played a pivotal role in achieving optimal resolution through hydrogen bonding interactions with the amino group of the adsorbent [29]. This detailed understanding of the molecular interactions involved highlights the intricate interplay between the chiral selector and the enantiomers, underscoring the importance of chromatographic conditions in achieving effective enantioseparation.

### Modeling Results

**Figure 4:**
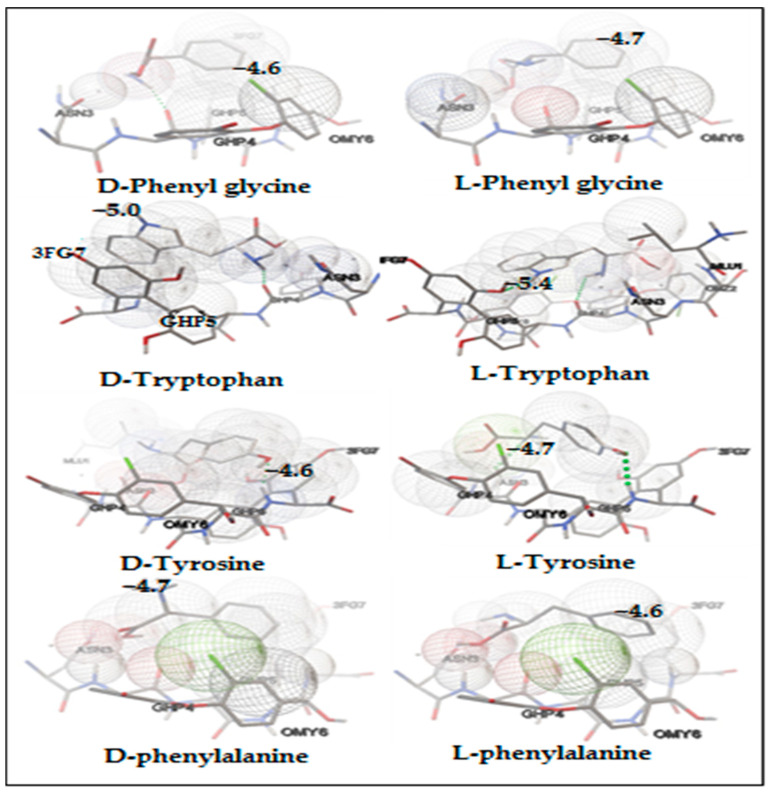
Three-dimensional model of interactions between amino acid enantiomers as ligands and Vancomycin chiral selector as receptor, generated using Autodock Vina software.

The primary objective of the docking study was not to precisely predict the ΔG (change in Gibbs free energy) of binding for individual molecules. Instead, this study was designed to facilitate relative comparisons of docking scores, which act as approximations of ΔG and are derived using a hybrid scoring function [30,31]. These docking scores were employed to forecast the elution order of each enantiomer and to analyze the binding modes, as well as the specific types of interactions that occur between the enantiomers and the chiral selectors [32,33]. The resulting energy profiles from the docking simulations, along with the lowest binding energy values observed between Vancomycin and the amino acid enantiomers, are detailed in the subsequent tables. This approach provides a comprehensive understanding of the molecular interactions and helps in predicting the behavior of the enantiomers during the separation process [34]. Additionally, Figure 4 illustrates 3D docking images depicting the interactions between amino acid enantiomers and Vancomycin, generated using AutoDockVina 1.1.2 software.

The molecular docking analysis offers invaluable insights into the intricate interactions between molecules, elucidating crucial aspects such as binding affinities, conformational preferences, and various intermolecular interactions [20]. By employing this comprehensive computational approach, researchers can gain a deeper understanding of the molecular mechanisms that underlie chiral recognition and separation processes [35]. The detailed analysis of molecular docking results enables us to determine the complex relationships between chiral selectors and the enantiomers of amino acids. This involves a thorough examination of the binding modes, energy landscapes, and specific interactions that govern these relationships [35,36]. Through such detailed computational investigations, we can better comprehend the factors that influence chiral separation and improve the design of more effective chiral selectors and separation techniques.

**Figure 5:**
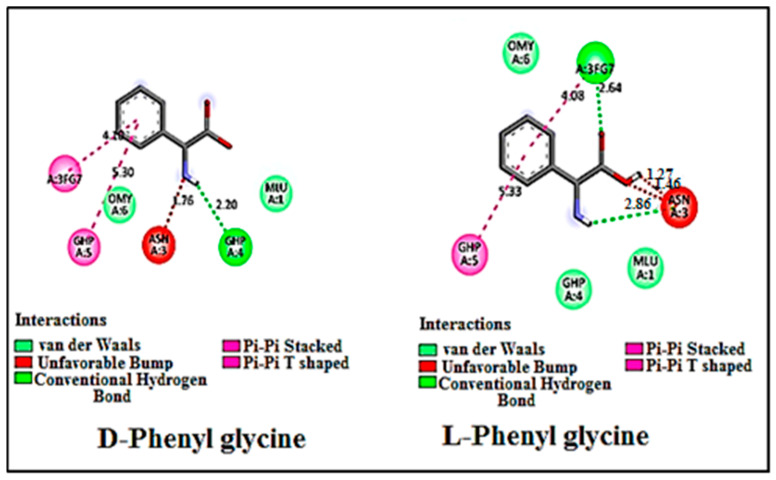
Images generated using Discovery Studio 21.1 Visualizer showing Vancomycin interacting with Phenylglycine enantiomers as ligands. The two enantiomers exhibit similar interactions with Vancomycin, with both enantiomers experiencing unfavorable binding interactions.

The molecular docking analysis revealed that both enantiomers of the racemic phenylglycine exhibited comparable ΔG values when interacting with the chiral selector, as depicted in Table 2. Furthermore, they adopted similar conformations within the Vancomycin selector, as illustrated in Figure 4. This similarity in ΔG values and conformations indicates that both enantiomers establish comparable intermolecular interactions, including Van der Waals, hydrogen bonding, and π-π interactions, as depicted in Figure 5. Consequently, these findings provide a rationale for the observed poor chromatographic resolution of phenylglycine amino acid when employing vancomycin as a chiral selector.

For both tryptophan and tyrosine, the binding energies varied between enantiomers within the same mixture, as depicted in Table 3 and Table 4. The magnitude of |ΔΔG| suggests that Vancomycin can effectively serve as a chiral selector for separating the enantiomers of tryptophan and tyrosine, aligning with the findings from chromatographic data. Specifically, the absolute value of |ΔΔG| for tryptophan exceeds that of tyrosine, thereby rationalizing the observed superior resolution of tryptophan compared with tyrosine resolution.

The complex formed between Vancomycin and D-tryptophan exhibited a lower |ΔG| value compared with that of L-tryptophan and Vancomycin, suggesting that D-tryptophan will elute first from the column. This phenomenon arises from the weaker stability of the complex formed between D-tryptophan and Vancomycin, as opposed to the stronger stability observed with L-tryptophan. This observation is consistent with the experimental results, wherein D-tryptophan displayed a shorter retention time compared with L-tryptophan.

**Figure 6:**
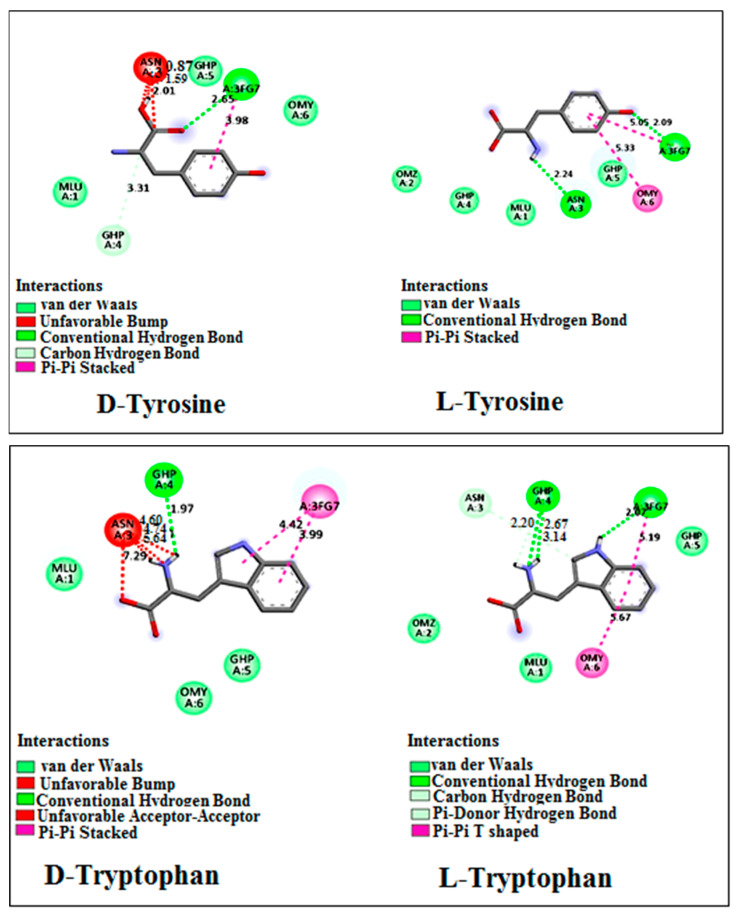
Two-dimensional images generated using Discovery Studio 21.1 Visualizer showing Vancomycin amino acids involved in interactions with tryptophan and tyrosine enantiomers as ligands. The interactions between Vancomycin and the enantiomers differ, with unfavorable binding occurring in the first elution enantiomer.

The diminished stability of the D-tryptophan-Vancomycin complex, relative to that of L-tryptophan, can be attributed to an unfavorable interaction between the amino acid moiety of Vancomycin and the amino group and acid group of D-tryptophan, as illustrated in Figure 6.

The L-tyrosine, with a final intermolecular energy of –5.41 kcal/mol, exhibits stronger interaction with the Vancomycin chiral selector compared with D-tyrosine, which has a final intermolecular energy of −4.94 kcal/mol. Consequently, a higher chromatographic retention time was observed for L-tyrosine. Figure 6 illustrates that L-tyrosine forms tighter interactions with Vancomycin than D-tyrosine, primarily due to the unfavorable interaction between the acid group of D-tyrosine and Vancomycin.

The poor resolution of phenylalanine enantiomers by the Vancomycin selector cannot solely be attributed to similar conformations inside the Vancomycin selector, as depicted in Figure 4. The docking analysis reveals that the (L)-enantiomer forms stronger binding interactions with the chiral selector. This is evident from the higher |ΔG| value of 4.34 kcal/mol, which represents the sum of van der Waals, hydrogen bonding, and desolvation energies, compared with the corresponding value for the (D)-enantiomer as indicated in Table 5. However, it is noteworthy that the electrostatic energy between the (D)-enantiomer and Vancomycin is greater than that between the chiral selector and the (L)-enantiomer.

**Figure 7:**
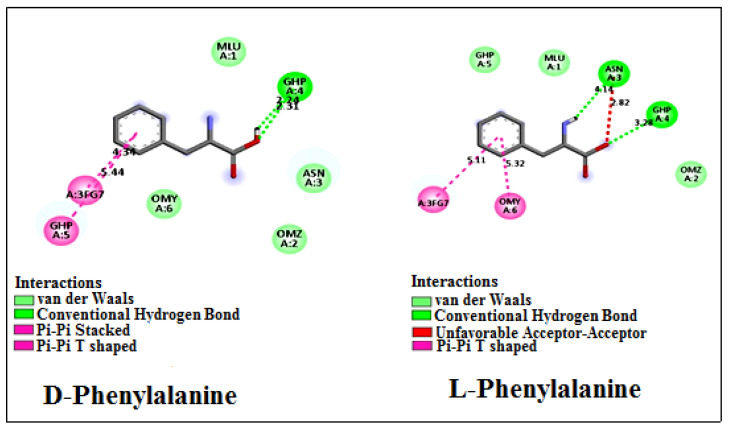
Two-dimensional images generated using Discovery Studio 21.1 Visualizer showing Vancomycin amino acids involved in interactions with phenylalanine enantiomers as ligands. The types and sites of interactions between Vancomycin and the enantiomers are notably different.

Although the docking poses of the enantiomer mixture differ, as shown in Figure 7, both enantiomer-Vancomycin complexes exhibit similar final intramolecular energy, as indicated in Table 5. Consequently, the docking analysis reveals a minimal difference in binding affinity between the enantiomers and Vancomycin, with a |ΔΔG| value of 0.24 kcal/mol. This finding elucidates the poor chromatographic separation observed for phenylalanine amino acid when utilizing Vancomycin as a chiral selector. It underscores the notion that the absence of interactions between the chiral selector and the enantiomers is not always the sole cause of poor resolution.

## 3. Materials and Methods

The analysis was conducted utilizing a Waters Alliance e2695 HPLC system (Waters corporation, Milford, MA, USA) equipped with a 2998 PDA detector, alongside Empower 3 software for data processing. Supporting instrumentation included a Sartorius semi-micro analytical balance model bp121s (Sartorius AG, Goettingen, Germany), a Digital pH meter from Mettler Toledo (Mettler-Toledo Gmbh, Greifensee, Schweiz), and an ultrasonic cleaner ANA. AU-32 HUMEAU (Humeau laboratories, Couëron, France). The chromatographic separation was carried out using an NH_2_ column (250 mm × 4.6 mm) manufactured by Merck KGaA (Darmstadt, Germany).

Racemic mixture amino acids and enantiomers were sourced from Sigma Aldrich (Hambourg, Germany), while all reagents utilized in this study, including methanol, 2-propanol, potassium hydroxide (KOH), and potassium dihydrogen phosphate (KH_2_PO_4_), were of analytical-reagent grade and procured from Sigma Aldrich.

### 3.1. Procedure 

A phosphate buffer solution (PBS) of 0.05 M was prepared by dissolving potassium dihydrogen phosphate and adjusting the pH to 6 using 0.1 M potassium hydroxide. Vancomycin stock solutions were then prepared by dissolving Vancomycin in PBS, followed by ultrasonic degasification. The mobile phase was prepared by mixing the buffer solution (pH 6.0) with 2-propanol in a 50/50 (*v*/*v*) ratio [37]. 

The analytes, comprising aromatic amino acids (tryptophan, phenylalanine, phenyl glycine, and tyrosine), were dissolved in methanol. Chromatographic separation was carried out with a flow rate of 0.8 mL/min and an injection volume of 10 μL. The influence of Vancomycin concentration in the mobile phase on chiral separation was investigated by varying the Vancomycin concentration within the range of 0–2.0 mM [38]. 

### 3.2. Molecular Docking Simulation

The molecular docking simulations were performed using an Intel(R) Core(TM) i3-6100U CPU @ 2.30GHz running Windows 10 operating system. This study encompassed three primary phases: (i) preparation of receptor and ligand pdb files involving database extension programming, (ii) execution of the molecular docking simulations, and (iii) analysis of the resulting data [39]. 

Initially, pdb files of Vancomycin and the enantiomers of tryptophan, tyrosine, phenylalanine, and phenylglycine were acquired. The pdb file of Vancomycin was sourced from the Protein Data Bank (www.rcsb.org) [40],while the enantiomeric pdb files were obtained from https://pubchem.ncbi.nlm.nih.gov/ (accessed on 25 February 2024) [41].

The Vancomycin pdb files were first loaded into AutoDock Tools (ADT) 4.2 to incorporate non-polar hydrogen atoms, followed by Gasteiger charge calculation. Once all necessary adjustments were made for the docking study, the Vancomycin pdb files were saved in pdbqt format [42]. Subsequently, each pdb file of the ligands (enantiomers of amino acids) was individually converted into pdbqt format using AutoDock Tools (ADT) 4.2. Docking was conducted using ADT, wherein all rotatable bonds of the ligands were considered as such, while the receptor was held rigid [43]. A grid box size of 44 × 40 × 36 Å with a spacing of 0.519 Å was utilized.

The pdbqt formatted files of the enantiomers were then docked with the targets one by one employing the AutoDock Vina program [44]. Targeted docking was employed, with the source coordinates set at X = 21.8, Y = 19.98, and Z = 21.8. Fifty independent docking runs were executed for each ligand (enantiomer) and target to identify the lowest free energy binding conformation from the largest cluster.

Upon saving both files in pdbqt format, Vina 1.1.2 software was utilized to compute the binding energy/affinity between the Vancomycin receptor and ligand (enantiomer). Subsequently, the output file was imported into AutoDock for virtual screening, molecular docking, and binding site analysis to generate images illustrating interactions and hydrogen bond lengths between Vancomycin amino acids and enantiomers. Two-dimensional schematic representations of the docking results were generated using Discovery Studio Visualizer [45,46].

## 4. Conclusions 

This study highlights the chiral separation of aromatic amino acids, taking advantage of Vancomycin as a chiral additive in the mobile phase and an achiral amino column as the stationary phase. Utilizing Autodock Vina software, molecular docking simulations were conducted between amino acid enantiomers as ligands and Vancomycin as the receptor across various targets. These simulations aimed to assess binding affinities, showcase the feasibility of resolution for enantiomers, and elucidate the mechanisms underpinning chiral recognition.

The findings revealed the efficacy of employing Vancomycin as a chiral additive in the mobile phase, paired with an NH_2_ column as the stationary phase, for separating the racemic mixture of tryptophan and tyrosine. This chromatographic condition demonstrated superior resolution compared with prior literature findings, especially when compared to using Vancomycin as a chiral stationary phase or other stationary phases for tryptophan and tyrosine enantiomer separation. However, this method proved inadequate for separating the enantiomeric mixture of phenylglycine and phenylalanine.

Furthermore, molecular docking simulations were utilized to illustrate host-guest interaction models between Vancomycin selectors and enantiomers. These models offered insights into the interactions influencing enantioseparation, elucidated the elution order of enantiomers, and provided explanations for the poor chiral resolution of phenylglycine and phenylalanine racemic mixtures. Notably, hydrogen interactions, hydrophobic interactions, and π–π exchanges emerged as the primary mechanisms driving the chiral recognition of Vancomycin selectors with different enantiomers. These findings suggest that while poor chiral separation is not solely attributable to the absence of interactions between selectors and enantiomers, it can also stem from similar final energy interactions between enantiomers as ligands and the chiral selector as the receptor.

## Figures and Tables

**Table 1:** Retention factor, selectivity, and resolution of amino acids using different concentrations of Vancomycin as a chiral additive in the mobile phase.

Amino Acids	Concentration of Chiral Selector (mM)	Retention Factor	Selectivity	Resolution
Phenylglycine	0	5.075		
	0.5	5.073	--------	--------
	1.0	5.051	--------	--------
	1.5	5.023	--------	--------
	2.0	5.011	--------	--------
Phenylalanine	0	5.183		
	0.5	5.175	--------	--------
	1.0	5.184	--------	--------
	1.5	5.197	--------	--------
	2.0	k_1_ = 5.153	1.01	--------
		k_2_ = 5.247		
Tryptophan	0	6.99		
	0.5	k_D_ = 5.259	1.44	2.93
		k_L_ = 7.497		
	1.0	k_D_ = 5.201	1.45	3.09
		k_L_ = 7.552		
	1.5	k_D_ = 5.155	1.68	3.98
		k_L_ = 8.663		
Tyrosine	0	5.035	--------	--------
	0.5	k_D_ = 6.480	1.19	1.89
		k_L_ = 7.724		
	1.0	k_D_ = 7.180	1.25	2.04
		k_L_ = 9.041		
	1.5	k_D_ = 8.743	1.50	2.97
		k_L_ = 13.182		

**Table 2:** Docking results of energy types and lowest binding energy between Vancomycin and phenylglycine enantiomers using Autodock Vina 1.1.2 software.

**Phenylglycine**	**Type of Energy (kcal/mol)**	**ΔG_D_**	**ΔG_L_**	**|ΔΔG|**
	Estimated Free Energy of Binding	−3.19	−3.12	0.03
	Final Intermolecular Energy	−4.38	−4.31	0.07
	vdW + Hbond + desolv Energy	−4.08	−3.91	0.17
	Electrostatic Energy	−0.30	−0.40	0.10
	Final Total Internal Energy	−1.59	−1.72	0.13
	Torsional Free Energy	+1.19	+1.19	
	Unbound System’s Energy	−1.59	−1.72	0.13

**Table 3:** Docking results of energy types and lowest binding energy between Vancomycin and tryptophan enantiomers using AutoDock Vina 1.1.2 software.

**Tryptophan**	**Type of Energy (kcal/mol)**	**ΔG_D_**	**ΔG_L_**	**|ΔΔG|**
	Estimated Free Energy of Binding	−3.73	−4.50	0.77
	Final Intermolecular Energy	−5.23	−5.99	0.76
	vdW + Hbond + desolv Energy	−4.83	−5.41	0.58
	Electrostatic Energy	−0.40	−0.58	0.18
	Final Total Internal Energy	−2.24	−1.95	0.29
	Torsional Free Energy	+1.49	+1.49	
	Unbound System’s Energy	−2.24	−1.95	0.29

**Table 4:** Docking results of energy types and the lowest binding energy from Vancomycin with tyrosine enantiomers using AutoDock Vina 1.1.2 software.

**Tyrosine**	**Type of Energy (kcal/mol)**	**ΔG_D_**	**ΔG_L_**	**|ΔΔG|**
	Estimated Free Energy of Binding	−3.15	−3.62	0.49
	Final Intermolecular Energy	−4.94	−5.41	0.47
	vdW + Hbond + desolv Energy	−4.59	−5.94	0.35
	Electrostatic Energy	−0.35	−0.47	0.12
	Final Total Internal Energy	−2.05	−1.83	0.22
	Torsional Free Energy	+1.49	+1.49	
	Unbound System’s Energy	−2.05	−1.83	0.22

**Table 5:** Docking results of energy types and the lowest binding energy from Vancomycin with phenylalanine enantiomers using AutoDock Vina software.

**Phenylalanine**	**Type of Energy (kcal/mol)**	**ΔG_D_**	**ΔG_L_**	**|ΔΔG|**
	Estimated Free Energy of Binding	−3.00	−3.24	0.24
	Final Intermolecular Energy	−4.49	−4.73	0.24
	vdW + Hbond + desolv Energy	−3.68	−4.34	0.66
	Electrostatic Energy	−0.81	−0.39	0.42
	Final Total Internal Energy	−2.15	−1.92	0.23
	Torsional Free Energy	+1.49	+1.49	
	Unbound System’s Energy	−2.15	−1.92	0.23

## Data Availability

Data are contained within the article.
